# Rethinking Adaptive Contextual Information and Multi-Scale Feature Fusion for Small-Object Detection in UAV Imagery

**DOI:** 10.3390/s25237312

**Published:** 2025-12-01

**Authors:** Chang Liu, Yong Wang, Qiang Cao, Changlei Zhang, Anyu Cheng

**Affiliations:** 1 Intelligent Manufacturing and Automobile School, Chongqing Polytechnic University of Electronic Technology, Chongqing 401331, China; 2 School of Automation, Chongqing University of Posts and Telecommunications, Chongqing 400065, China

**Keywords:** UAV, small object detection, contextual information, cross-scale fusion

## Abstract

Small object detection in unmanned aerial vehicle (UAV) imagery poses significant challenges due to insufficient feature representation, complex background interference, and extremely small target sizes. These factors collectively degrade the performance of conventional detection algorithms, leading to low accuracy, frequent missed detections, and false alarms. To address these issues, we propose **YOLO-DMF**, which is a novel detection framework specifically designed for drone-based scenarios. Our approach introduces three key innovations from the perspectives of feature extraction and information fusion: (1) a **D**etail-Semantic Adaptive Fusion (DSAF) module that employs a multi-branch architecture to synergistically enhance shallow detail features and deep semantic information, thereby significantly improving feature representation for small objects; (2) a **M**ulti-Scale Residual Spatial Attention (MSRSA) mechanism incorporating scale-adaptive spatial attention to improve robustness against background clutter while enabling a more precise localization of critical target regions; and (3) a **F**eature Pyramid Reuse and Fusion Network (FPRFN) that introduces a dedicated 160×160 detection head and hierarchically combines multi-level shallow features with high-level semantic information through cross-scale fusion, effectively enhancing sensitivity to both small and tiny objects. Comprehensive experiments on the VisDrone2019 dataset demonstrate that YOLO-DMF outperforms state-of-the-art lightweight detection models. Compared to the baseline YOLOv8s, our method achieves improvements of 3.9% in mAP@0.5 and 2.5% in mAP@0.5:0.95 while reducing model parameters by 66.67% with only a 2.81% increase in computational cost. The model achieves a real-time inference speed of 34.1 FPS on the RK3588 NPU, satisfying the latency requirements for real-time object detection. Additional validation on both the AI-TOD and WAID datasets confirms the method’s strong generalization capability and promising potential for practical engineering applications.

## 1. Introduction

With the rapid advancement of high-resolution imaging, wireless communication, and sensor technologies, unmanned aerial vehicles (UAVs) have been widely applied in urban traffic management, agriculture, power line inspection, and military reconnaissance [1]. However, due to the high-altitude, long-distance imaging perspective, targets in UAV-captured images are typically small, densely distributed, and suffer from weak features, severe occlusion, and complex background clutter [2], posing significant challenges for small object detection.

Traditional object detection algorithms primarily rely on hand-crafted features such as the Histogram of Oriented Gradients (HOG) [3] and Scale-Invariant Feature Transform (SIFT) [4], which are typically combined with sliding window mechanisms for multi-scale region proposal generation. The extracted features are then fed into classifiers like Support Vector Machines (SVMs) [5] for object recognition. However, these conventional methods suffer from limited feature representation due to their dependence on manually designed features, resulting in poor generalization capability and robustness, which severely restricts their effectiveness in complex small object detection scenarios. In contrast, deep learning-based models automatically learn hierarchical feature representations from data, overcoming the limitations of hand-crafted designs. Among them, one-stage detectors such as YOLO [6] and RetinaNet [7] directly regress bounding boxes with high inference speeds (tens to hundreds of frames per second), offering a favorable balance between accuracy and efficiency—making them more suitable for real-time deployment on resource-constrained UAV platforms.

Most existing object detection methods, designed for general scenarios, perform poorly on UAV images due to the challenges of small object scale, dense distribution, and complex background interference. To address these issues, Liu et al. [8] proposed YOLC, which is a UAV small object detection algorithm that significantly improves performance through a Local Scale Module (LSM), Gaussian Wasserstein Distance (GWD) regression loss, and detection head refinement with deformable convolutions. Song et al. [9] developed GLF-Net for rotated small object detection in UAV images, employing a flip- and self-attention-based backbone network with multi-scale feature extraction to enhance the perception of rotated small objects. Lin et al. [10] proposed HawkNet, which improves small object detection through an expanded feature aggregation framework and novel upsampling methods. Ye et al. [11] introduced RTD-Net, an efficient real-time object detection network for UAV images, incorporating a Feature Fusion Module (FFM), Lightweight Extraction Module (LEM), and Convolutional Multi-Head Self-Attention (CMHSA) mechanism.

Despite significant progress, current methods generally handle fine-grained details and high-level semantics separately, even though they enhance feature learning through improved network modules, optimized fusion strategies, or attention mechanisms. They often rely on static fusion rules that fail to account for the spatial sparsity and structural fragility of tiny objects. As a result, critical spatial cues are progressively degraded in deep network layers, while cluttered background regions may be mistakenly activated as object-like responses. Consequently, state-of-the-art detectors still suffer from high miss rates and frequent false alarms in complex, multi-scale UAV scenarios. This underscores the need for a more discriminative and adaptive feature learning paradigm capable of jointly optimizing detail preservation, semantic enrichment, and background suppression to achieve robust small object detection in aerial imagery. To address these challenges, we propose YOLO-DMF, a novel small object detection algorithm specifically designed for UAV scenarios, with three key contributions:

(1) A DSAF module that enhances contextual information representation by effectively integrating detailed features with semantic information, thereby improving the feature representation of small objects.
(2) An MSRSA mechanism that overcomes the limitations of traditional spatial attention in multi-scale modeling and adaptability, effectively mitigating interference from complex backgrounds and scale variations.
(3) An FPRFN that incorporates a dedicated 160×160 detection head to enhance tiny object perception, along with a multi-level feature reuse strategy and cross-scale fusion of high-level semantic information, significantly improving performance in small object detection tasks.

## 2. Related Work

### 2.1. Contextual Information Modeling

In small object detection tasks, the inherently limited pixel coverage and minimal feature representation of targets make it challenging to achieve accurate localization and recognition when relying solely on local features. Consequently, effectively modeling and leveraging contextual information has emerged as a critical research direction for improving detection performance. Contextual information, which includes spatial layouts, semantic relationships, and interactions between objects in the surrounding environment, offers valuable supplementary cues that improve overall detection performance [12]. This broader perspective enables the model to better understand and identify small targets by considering their relationship with the visual context.

Recent studies have incorporated contextual modeling mechanisms to improve detector robustness. Some approaches employ dilated convolutions [13] or non-local operations [14] to expand receptive fields and capture broader contextual information. Others design specialized context enhancement modules, such as global attention mechanisms [15] or graph neural networks (GNNs) [16], to dynamically filter and emphasize relevant contextual regions. These strategies have demonstrated significant improvements in detection accuracy and stability, particularly in complex backgrounds.

Moreover, in UAV aerial imagery and remote sensing applications, small objects often appear densely distributed amidst severe background clutter, where inadequate contextual modeling frequently leads to false positives and missed detections. To address this, researchers have developed various context-aware modules. For instance, Liang et al. [17] proposed a feature fusion-based single-shot detector (FS-SSD) incorporating spatial context analysis for multi-category small object detection in UAV images. Similarly, Han et al. [18] introduced a context-scale-aware detector (CSADet) that jointly exploits local and global contextual cues to enhance performance.

Existing studies have demonstrated that context modeling can enhance small object detection accuracy in UAV applications to some extent. However, current context modeling mechanisms still exhibit notable limitations, particularly in achieving adaptive fusion between fine-grained detail features and high-level semantic information. To address these challenges, future research should prioritize the development of more sophisticated adaptive context modeling approaches. Key research directions should focus on investigating effective strategies for context information fusion in complex and dynamic scenarios with the ultimate goal of significantly improving both the precision and robustness of small object detection systems.

### 2.2. Multi-Scale Feature Fusion

In small object detection, significant variations in object scales and weak feature representations are among the key factors limiting detection performance. Multi-scale feature fusion techniques, which integrate hierarchical feature information, have emerged as a pivotal solution to this challenge. By combining fine-grained texture details from shallow layers with high-level semantic information from deep layers, these methods enhance the model’s capability to perceive small objects.

Current mainstream object detection frameworks predominantly employ Feature Pyramid Networks (FPNs) [19] and their variants (e.g., PANet [20], BiFPN) for multi-scale feature fusion. These architectures leverage top–down and lateral connections to propagate high-level semantic features to lower-level feature maps, thereby strengthening the semantic representation of small objects. Experimental results demonstrate that such fusion strategies significantly improve detection performance for small objects in complex backgrounds. Recent work augments FPN with attention and skip links for better multi-scale fusion.

Existing multi-scale feature fusion methods have been widely applied in small object detection tasks based on UAV imagery, demonstrating promising performance in enhancing the robustness of models to scale variations. For example, Zhang et al. [21] proposed an innovative Multi-Scale and Occlusion-Aware Network (MSOA-Net), which incorporates an adaptive feature fusion module to dynamically aggregate multi-level features, effectively handling scale variations of vehicles from UAV perspectives. Similarly, Qiu et al. [22] introduced an Adaptive Spatial Feature Fusion strategy (ASFF-YOLOv5), leveraging Receptive Field Blocks (RFBs) to enhance scale-invariant feature learning, which is specifically tailored for multi-scale traffic element detection in UAV imagery. The RSUD20K dataset introduced by Zunair et al. [23], characterized by diverse geographical scenes, heavy occlusions, and objects exhibiting extreme scale variations, highlights the limitations of current object detection methods in effectively performing multi-scale feature fusion.

However, current approaches often overlook two critical aspects of cross-level feature fusion: the disparity in feature map density and the inconsistency in semantic information. Significant semantic gaps exist between features at different scales, and their contributions to small object detection can vary considerably. A simple or naive fusion strategy may undermine the model’s ability to represent multi-scale targets, ultimately degrading the overall detection performance. Future research should therefore focus on developing lightweight multi-scale fusion modules that not only preserve fine-grained details during feature propagation but also achieve semantically coherent integration across scales.

## 3. Proposed Method

### 3.1. Overall Architecture

The YOLOv8 architecture comprises three core components: a backbone network for feature extraction, a neck network for multi-scale feature fusion, and a head module for object detection. The backbone employs lightweight C2f modules to replace the CSPBlock structures in earlier YOLO versions, incorporating an enhanced Spatial Pyramid Pooling Fast (SPPF) module that improves both feature extraction efficiency and global feature perception. The neck section maintains the PANet and FPN fusion strategy while substituting traditional C3 structures with C2f modules and simplifying upsampling paths, achieving more efficient and direct multi-scale feature integration. The detection head adopts an anchor-free decoupled design that separately models classification and bounding box regression tasks, eliminating redundant objectness branches to enhance detection accuracy while significantly accelerating inference speed. Furthermore, YOLOv8 offers five configurations (n, s, m, l, x) with varying network depths and widths to accommodate different computational constraints and accuracy requirements.

Considering the resource limitations of UAV platforms and the high detection difficulty of the VisDrone dataset, using the lightest YOLOv8n model yields suboptimal results, while larger models show limited performance gains despite substantially increased parameters and computational complexity. Therefore, this study selects YOLOv8s as the baseline model after comprehensive evaluation of the performance–resource trade-off.

**Figure 1:**
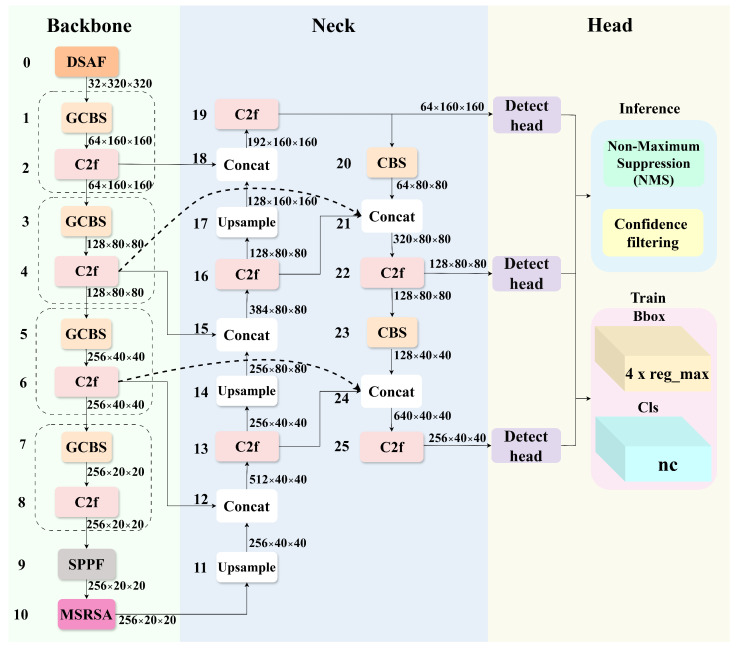
Network architecture of YOLO-DMF. GCBS denotes Group Convolution, Batch Normalization, and SiLU activation function.

To enhance detection performance in complex UAV scenarios, we introduce three key improvements to the YOLOv8s architecture. First, the first conventional strided convolution in the backbone is replaced with a DSAF module, which facilitates the effective integration of low-level detail features and high-level semantic information through multi-branch feature interaction. Second, an MSRSA module is incorporated after the SPPF block, aiming to improve target localization accuracy under complex background interference and enhance robustness to multi-scale noise. Third, we design a FPRFN to address the spatial detail loss caused by repeated downsampling. This module enables the optimized reconstruction of multi-level feature maps through cross-layer feature reuse and adaptive fusion mechanisms. The overall architecture and detailed module configurations of the proposed YOLO-DMF model are illustrated in Figure 1 and Table 1.

### 3.2. DSAF Module

In UAV-based small object detection tasks, conventional detection methods often fail to adequately capture subtle target features due to the inherently small size of objects, low visual saliency, and complex background interference, which ultimately limits detection accuracy. This challenge originates from two fundamental issues: (1) Successive downsampling operations in deep networks progressively erode fine-grained details of small objects, compromising their representational fidelity in deep feature maps and thereby degrading localization precision; and (2) although shallow features retain richer spatial details, their weaker semantic representation makes them more susceptible to background noise in complex environments, further exacerbating the difficulty of extracting discriminative features for small targets. These limitations highlight a critical research challenge: how to achieve an effective balance between fine-grained detail preservation and high-level semantic enhancement during feature extraction, enabling the model to holistically perceive both the target and its surrounding environment across different abstraction levels.

**Figure 2:**
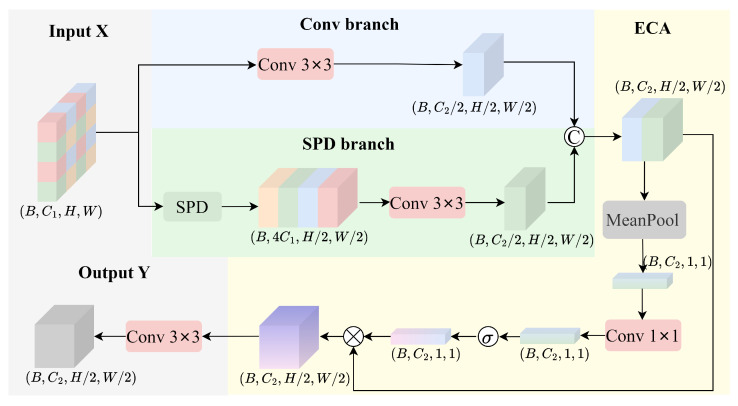
The structure of DSAF module.

To address this challenge, we propose a Detail-Semantic Adaptive Fusion (DSAF) module as illustrated in Figure 2. The DSAF module is composed of three core components: a convolutional branch (Conv branch), a spatial decomposition branch (SPD branch) [24], and an attention-enhanced mechanism. Through the adaptive fusion of hierarchical feature representations, this module effectively preserves fine-grained spatial details while enhancing semantic expressiveness. This balanced design significantly improves the model’s ability to represent and understand contextual information, which is particularly crucial for detecting small objects in complex scenes.

Let the input feature map be denoted as $X\in R^{B\times C_{1}\times H\times W}$, where $C_{1},H,$ and *W* represent the number of input channels, the height, and the width of the feature map, respectively.

First, a 3×3 convolution with stride 2 is applied to the input feature map X for spatial downsampling, which extracts high-level semantic information, resulting in feature map $F_{\mathrm{conv}}$.

(1) $$F_{\mathrm{conv}}=SiLU\left(BN\left(Conv_{k=3}^{s=2}\left(X\right)\right)\right)\in R^{B\times \frac{C_{2}}{2}\times \frac{H}{2}\times \frac{W}{2}}$$

To preserve spatial details and avoid the information loss caused by traditional downsampling methods, the spatial-preserving branch introduces a pixel rearrangement for detail operation. This operation rearranges 2×2 local regions from the input feature map into the channel dimension, effectively increasing the channel depth while maintaining spatial resolution. Subsequently, a 3×3 convolutional layer is applied to compress the channels, resulting in a high-resolution feature map $F_{\mathrm{SPD}}$ that retains detailed spatial information.

(2) $$X_{\mathrm{SPD}}=Concat\left(X_{::2,::2},X_{1::2,::2},X_{::2,1::2},X_{1::2,1::2}\right)\in R^{B\times 4C_{1}\times \frac{H}{2}\times \frac{W}{2}}$$

(3) $$F_{\mathrm{SPD}}=SiLU\left(BN\left(Conv_{k=3}^{s=1}\left(X_{\mathrm{SPD}}\right)\right)\right)\in R^{B\times \frac{C_{2}}{2}\times \frac{H}{2}\times \frac{W}{2}}$$

To further enhance the feature fusion effect, the module incorporates an Efficient Channel Attention (ECA) mechanism [25]. Specifically, the outputs from the semantic branch and the SPD branch are first concatenated along the channel dimension to obtain a fused feature map $F_{\mathrm{fuse}}$. Global average pooling is then applied to $F_{\mathrm{fuse}}$ to generate a channel-wise statistical descriptor, which captures global feature information for subsequent attention recalibration.

(4) $$F_{\mathrm{fuse}}=Concat\left(F_{\mathrm{Conv}},F_{\mathrm{SPD}}\right)\;\;\in R^{B\times C_{2}\times \frac{H}{2}\times \frac{W}{2}}$$

(5) $$z=\frac{1}{H\cdot W}\sum_{i=1}^{H}\sum_{j=1}^{W}F_{\mathrm{fuse}}\left[:,:i,j\right]$$

Subsequently, a 1×1 convolutional layer is employed to model the inter-channel dependencies, which is followed by a Sigmoid activation function that generates the channel-wise attention weights w. These weights are then used to dynamically modulate the fused feature map, enhancing informative channels while suppressing less relevant ones.

(6) $$w=\sigma (Conv_{k=1}\left(z\right))$$

(7) $$F_{\mathrm{att}}=F_{\mathrm{fuse}}⊙w$$

Finally, a 3×3 convolutional layer is applied to map the channel dimension back to the target dimension $C_{2}$, yielding the output feature map $Y\in R^{B\times \frac{C_{2}}{2}\times \frac{H}{2}\times \frac{W}{2}}$.

(8) $$Y=Conv_{k=3}^{s=1}\left(F_{\mathrm{att}}\right)$$

### 3.3. MSRSA Mechanism

In UAV-based object detection tasks, background noise interference constitutes one of the key factors limiting model performance. Since UAV-captured scenes typically involve complex ground textures, shadowed regions, uneven illumination, and dynamic environments affected by weather conditions, these factors collectively form multi-scale background noise sources. Such noise not only significantly increases the complexity of image backgrounds but also tends to cause confusion with the visual features of target objects, making this interference particularly pronounced.

In recent years, spatial attention mechanisms have been widely adopted in backbone networks and detection heads due to their remarkable ability to highlight critical regions while suppressing background interference, thereby significantly enhancing the model’s perception of target locations and boundaries. However, prevailing spatial attention modules (e.g., CBAM [26], SCSE [27]) typically rely on single-scale convolutions or fixed-receptive-field pooling operations to generate attention weights, which struggle to effectively model multi-scale targets and their semantic contexts in UAV-based small object detection tasks characterized by large-scale target variations and strong background interference. This lack of adaptivity limits their generalization capability for targets of diverse sizes and shapes in complex environments, preventing the full exploitation of attention mechanisms’ potential. Consequently, there is an urgent need to develop a spatially adaptive attention architecture with scale-awareness and dynamic adjustment capabilities to improve the model’s precision in localizing and enhancing features of small targets against cluttered backgrounds.

**Figure 3:**
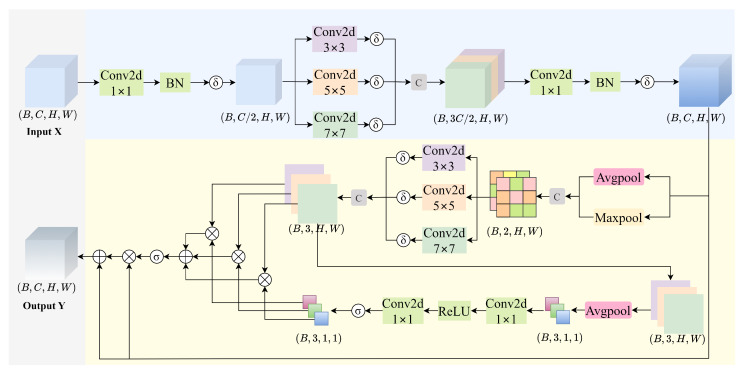
Illustration of MSRSA module. δ(·) represents the SiLU activation function, and σ(·) indicates the Sigmoid activation function.

To address the aforementioned limitations, we propose a lightweight yet effective Multi-Scale Residual Spatial Attention (MSRSA) mechanism, as illustrated in Figure 3. The module is designed to aggregate multi-scale spatial contextual information and generate more stable and discriminative spatial attention maps through a residual fusion strategy. This approach not only enhances the model’s ability to identify critical regions in complex scenes but also significantly improves robustness against multi-scale background noise, thereby overcoming key limitations of existing attention mechanisms in UAV-based small object detection.

Given an input feature map $X\in R^{B\times C_{1}\times H\times W}$, the channel dimension is first reduced by a compression factor γ (set to γ in this study) through a feature compression operation.

(9) $$X_{R}=SiLU\left(BN\left(Conv_{1\times 1}\left(X\right)\right)\right)\in R^{B\times \frac{C}{2}\times H\times W}$$

The compressed feature $X_{R}$ is then processed by parallel depthwise convolutions with varying kernel sizes $k_{i}\in \left\{3,5,7\right\}$.

(10) $$F_{k_{i}\times k_{i}}=SiLU\left(DepthwiseConv_{k_{i}\times k_{i}}\left(X_{R}\right)\right)$$

The multi-scale features $\left\{F_{3},F_{5},F_{7}\right\}$ are concatenated and fused via a 1×1 convolution to restore the original channel dimension.

(11) $$X_{\mathrm{ms}}=SiLU\left(BN\left(Conv_{1\times 1}\left(Concat\left(F_{3\times 3},F_{5\times 5},F_{7\times 7}\right)\right)\right)\right)\in R^{B\times C\times H\times W}$$

Next, a multi-scale spatial attention module enhances the spatial features through the following steps:

(a) Pooling concatenation: Combine average and max pooling outputs.
  (12) $$Z_{\mathrm{pool}}=Concat\left(AvgPool\left(X_{\mathrm{ms}}\right),MaxPool\left(X_{\mathrm{ms}}\right)\right)\in R^{B\times 2\times H\times W}$$
(b) Multi-scale convolution: Apply parallel convolutions to $Z_{pool}$.
  (13) $$M_{k_{i}\times k_{i}}=SiLU\left(Conv_{k_{i}\times k_{i}}\left(Z_{\mathrm{pool}}\right)\right),k_{i}\in \left\{3,5,7\right\}$$
(c) Attention fusion: Generate attention weights via adaptive average pooling (AAP) and Sigmoid activation.
  (14) $$A=\sigma \left(Conv_{1\times 1}\left(ReLU\left(Conv_{1\times 1}\left(AAP\left(Concat\left(M_{3\times 3},M_{5\times 5},M_{7\times 7}\right)\right)\right)\right)\right)\right)\in R^{B\times 3\times 1\times 1}$$

Finally, the output feature Y is obtained by a residual connection with attention-weighted feature refinement.

(15) $$Y=X_{\mathrm{ms}}+X_{\mathrm{ms}}⊙\sigma (\sum_{i=1}^{3}A_{i}\cdot M_{k_{i}\times k_{i}}),k_{i}\in \left\{3,5,7\right\}$$

### 3.4. FPRFN

**Figure 4:**
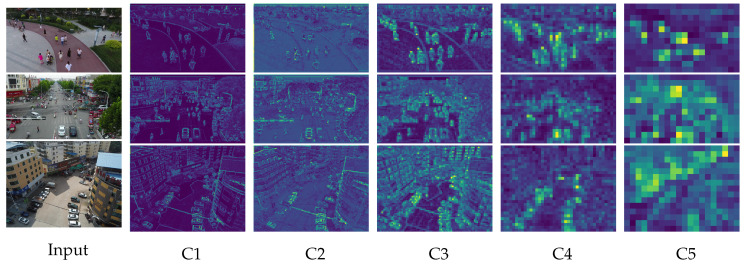
Visualization of feature maps across extraction layers.

In UAV-based object detection tasks, challenges such as long imaging distances, high-speed motion, and low-altitude flight often lead to motion blur, which results in small-sized targets with poor feature distinguishability, thereby degrading training efficiency and detection accuracy. Particularly when the UAV operates at higher altitudes, tiny objects in the captured images are prone to missed detection due to their extremely small scale. Although the original YOLOv8 architecture incorporates multi-scale feature maps (20×20, 40×40, and 80×80) to detect small- and medium-sized objects, it still exhibits limited capability in extracting discriminative features for tiny objects commonly encountered in UAV imagery. As the network depth increases, the resolution of deep convolutional feature maps progressively decreases. The coarse-grained semantic information in these layers contains a large amount of redundant data, causing fine-grained details of tiny targets to be gradually suppressed during the feature abstraction process (as illustrated in stages C4 and C5 in Figure 4). Consequently, such targets become indistinguishable from background noise, significantly degrading the model’s ability to perceive small objects. This fundamental limitation substantially compromises YOLOv8’s performance on drone-captured datasets where tiny objects predominate.

**Figure 5:**
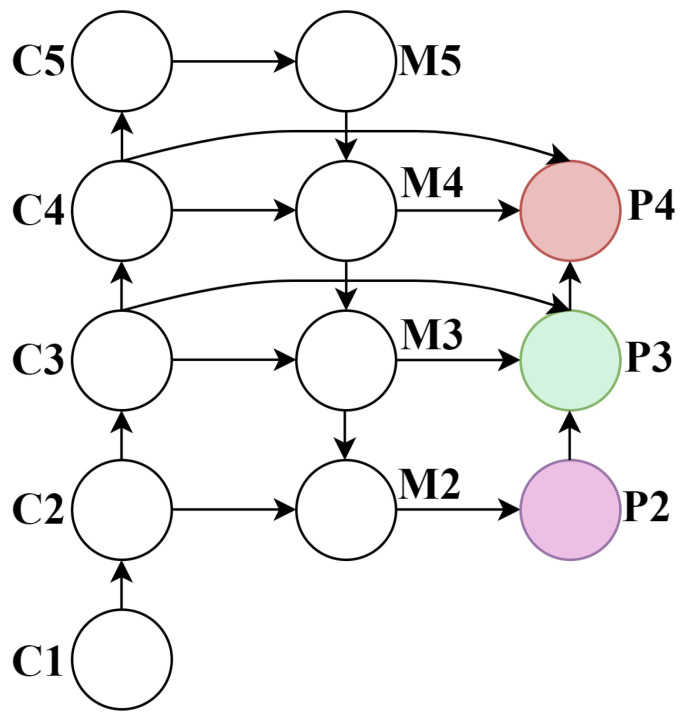
FPRRN network schematic. Here, C denotes the backbone feature maps, M represents the merged features after fusion in the neck, and P indicates the predictive features used for multi-scale detection.

To address this issue, we propose an enhanced neck architecture termed the Feature Pyramid Reuse and Fusion Network (FPRFN), as illustrated in Figure 5. Building upon the baseline model, we introduce a 160×160 detection head specifically designed for tiny objects. This head leverages high-resolution features extracted from shallower network layers, preserving the finer spatial details critical for small object recognition. Our modifications substantially improve the model’s capacity to learn discriminative features of minuscule targets, thereby mitigating missed detections in UAV scenarios.

Concurrently, we remove the original 20×20 detection head, which was designed for large objects. While this modification results in a marginal decrease in precision for large-scale targets, it effectively reduces the number of model parameters and accelerates training convergence, thereby enabling the model to focus more sharply on small object detection tasks.

Furthermore, to mitigate the loss of critical fine-grained details (e.g., edges and textures) caused by repeated downsampling in deep networks, we propose multi-level feature reuse from shallow layers of the backbone. These features are then fused with high-level semantic features at multiple scales, effectively compensating for the spatial information degradation induced by deep convolutions. This strategy not only enhances the robustness of feature representations but also significantly improves performance in small object detection.

## 4. Experiments and Results Analysis

### 4.1. Dataset

To validate the effectiveness and generalization capability of the proposed method in complex scenarios, extensive experiments were conducted on multiple public datasets. The primary experimental data were obtained from the VisDrone2019 dataset [28], which was released by the Machine Learning and Data Mining Laboratory at Tianjin University. This dataset encompasses diverse aerial scenes captured across 14 cities, including urban and rural environments under varying weather conditions and illumination changes. It comprises a total of 8629 images with 6471 for training, 548 for validation, and 1610 for testing. Detailed statistical information is presented in Table 2. The detection challenges of VisDrone2019 primarily include high object density, abundant small and tiny objects, severe inter-object occlusions, and imbalanced category distribution. These characteristics make it a highly valuable benchmark for research.

To further evaluate the generalization capability of the proposed method, additional experiments were conducted on the WAID [29] and AI-TOD datasets [30]. WAID is a large-scale, multi-class, high-quality aerial image dataset specifically designed for wildlife monitoring using UAVs. It consists of 14,375 UAV-captured images collected under diverse environmental conditions, and it is divided into a training set (10,056 images), a validation set (2873 images), and a test set (1437 images). The dataset covers six wildlife species across various habitat types. Detailed statistical information is provided in Table 3.

AI-TOD is a large-scale benchmark dataset specifically designed for tiny object detection in aerial imagery. It comprises a total of 28,036 images, which are divided into a training set (11,214 images), a validation set (2804 images), and a test set (14,018 images). The dataset covers eight common aerial object categories, including aircraft, vehicles, and pedestrians, with over 700,000 annotated instances. Detailed statistical information is provided in Table 4. Notably, AI-TOD presents significantly greater detection challenges compared to other aerial datasets. The average object size in AI-TOD is merely 12.8 pixels, which is substantially smaller than that of typical targets in comparable benchmark datasets. This characteristic imposes stricter requirements on the robustness and small-object recognition capabilities of detection algorithms, making AI-TOD an ideal testbed for evaluating advanced detection methods.

### 4.2. Experimental Setup and Evaluation Metrics

To validate the effectiveness of the proposed algorithm, we set up a dedicated experimental environment. The system was implemented on the CentOS 8.5.2 operating system with deep neural network models developed using the PyTorch1.9 framework. Detailed hardware specifications and software configuration are summarized in Table 5. To ensure a fair comparison across all experiments, we kept the hyperparameter settings consistent during training. The complete configuration details are summarized in Table 6.

To comprehensively evaluate the object detection performance of the proposed model, we employ three key metrics: mean average precision (mAP), parameter count (Params), and computational complexity measured in Giga Floating Point Operations (GFLOPs). mAP is computed as the average of the average precision (AP) values across all object categories, serving as a standard metric for detection accuracy by summarizing the area under the precision–recall curve. Parameter count indicates the model’s structural complexity and memory footprint, while GFLOPs quantify the computational cost, thereby providing insights into inference efficiency and deployment feasibility on resource-constrained platforms. Furthermore, to assess the model’s real-time inference capability on edge devices, we conduct experiments on the RK3588 platform. With an input resolution of 640 × 640, the model is quantized and compiled into an INT8-precision inference engine. The forward inference speed (FPS) of the core network is then measured in pure NPU mode, providing an objective evaluation of the model’s computational efficiency and hardware compatibility in typical deployment scenarios.

(16) $$Precision=\frac{TP}{TP+FP}$$

(17) $$Recall=\frac{TP}{TP+FN}$$

(18) $$AP=\int_{0}^{1}P(R)dR$$

(19) $$mAP=\frac{1}{K}\sum_{k\in K}AP(k)$$

Here, TP, FP, and FN represent the counts of true positives, false positives, and false negatives, respectively.

### 4.3. Comparison and Analysis of Key Improvements

#### 4.3.1. Comparison Experiments of Different Attention Mechanisms

To validate the effectiveness and superiority of the proposed MSRSA attention mechanism, we conducted comparative experiments by integrating various attention modules into the baseline YOLOv8s architecture. All models were rigorously evaluated on the VisDrone2019 test set with quantitative results comprehensively summarized in Table 7. The experimental results summarized in Table 7 illustrate the impact of incorporating different attention mechanisms on model performance. Compared to the original YOLOv8s model, the integration of attention mechanisms such as ECA, SimAM, and EMA resulted in varying degrees of mAP degradation. This observation highlights the inherent challenges in small-object detection: due to the limited pixel count in small targets, their feature representations are weak and highly susceptible to background interference. These mechanisms may fail to effectively focus on critical target features and instead erroneously emphasize background regions. The findings suggest that general-purpose attention approaches often yield suboptimal performance on tiny objects with some even degrading baseline detection accuracy due to a lack of specialized adaptation for small-object characteristics.

Therefore, for small object detection tasks, there is a pressing need to develop more precise and efficient attention mechanisms that can enhance feature representation while reliably capturing and amplifying critical target localization information. In contrast, the proposed MSAHA attention mechanism achieves superior performance with only minimal additional computational overhead — improving overall detection accuracy by 0.3% in mAP@0.5 and 0.2% in mAP@0.5:0.95, while demonstrating particularly strong adaptability and advantages in small object detection.

#### 4.3.2. Comparative Experiments on Shallow Feature Fusion Methods

**Figure 6:**
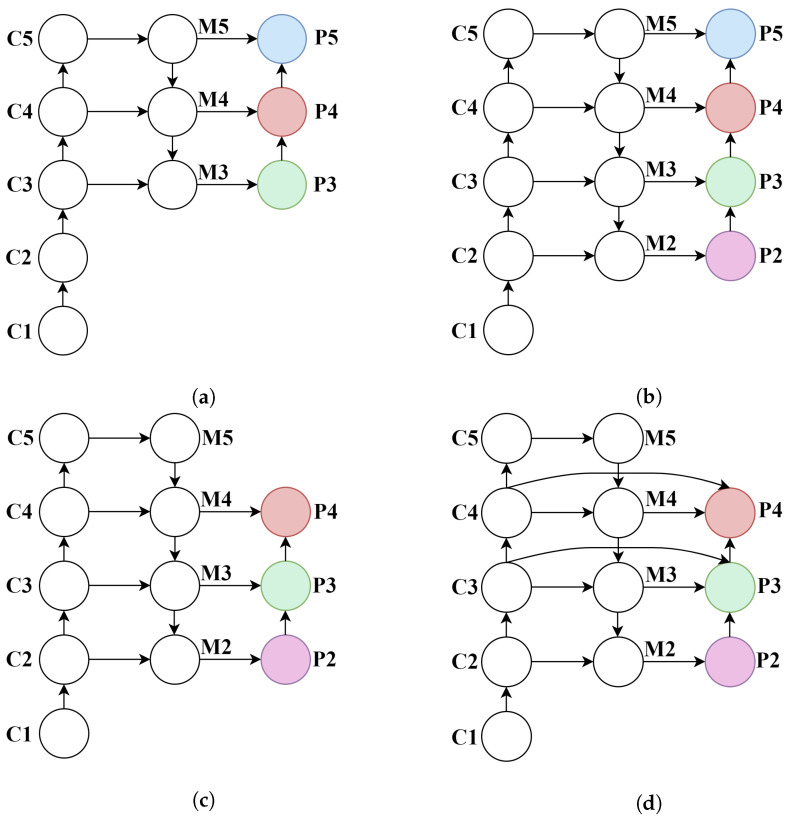
Comparative results of various feature fusion strategies. (**a**) PAN-P3+P4+P5. (**b**) PAN-P2+P3+P4+P5. (**c**) PAN-P2+P3+P4. (**d**) FPRFN-P2+P3+P4.

To validate the effectiveness of the proposed FPRFN in multi-scale feature fusion, we conducted comparative experiments using various feature fusion strategies. The experiments primarily focused on replacing the high-level feature layer P5 (as shown in Figure 6a), originally used for large object detection, with the shallow feature layer P2 (as shown in Figure 6b,c), which is more suitable for small object detection. In addition, different multi-scale feature fusion architectures, including PAN and the proposed FPRFN (as shown in Figure 6d), were evaluated. All experiments were tested and evaluated on the VisDrone2019 test dataset with the relevant results summarized in Table 7 and Table 8.

As shown in Table 8, the original YOLOv8s model employs a PAN with P3+P4+P5 as its neck structure for multi-scale feature fusion, achieving an mAP@0.5 of 31.4% on the test set. When the high-level feature layer P5 is replaced with the higher-resolution shallow feature layer P2, forming a P2+P3+P4 PAN structure, the model exhibits significant performance improvement in small object detection, with mAP@0.5 reaching 34.5%, and precision and recall increasing to 45.4% and 36.2%, respectively. These results demonstrate that incorporating high-resolution shallow features effectively enhances the model’s ability to perceive and represent the fine-grained details of small objects.

However, when integrating all four hierarchical features (i.e., adopting a P2+P3+P4+P5 PAN structure), the detection performance experiences a slight decline. This suggests that excessive multi-level feature fusion may introduce redundant information, interfering with the feature learning process for small objects and consequently impairing the overall detection efficacy. In contrast, the proposed FPRFN, based on P2+P3+P4 fusion, demonstrates superior architectural design and enhanced practicality for multi-scale feature integration. While maintaining reasonable computational complexity, this approach elevates mAP@0.5 to 34.8%, achieving an optimal balance between feature richness and detection efficiency.

Furthermore, as shown in Table 9, the incorporation of the P2 feature layer significantly enhances small object detection performance. The proposed FPRFN architecture demonstrates particularly notable improvements, achieving a 2.1% increase in the $AP_{s}$ (average precision for small objects) metric, along with simultaneous gains of 1.4% in $AP_{M}$ (medium objects) and 0.7% in $AP_{L}$ (large objects).

The above experimental results fully validate the effectiveness of the FPRFN in network architecture design, particularly highlighting its superior performance in small-object detection tasks. These findings underscore its strong potential for practical engineering applications and real-world deployment.

### 4.4. Ablation Experiments

To systematically evaluate the impact of each improvement module on small object detection performance, we designed a series of ablation experiments by progressively incorporating different modules into the YOLOv8s framework. The investigated improvement modules include the DSAF module, the MSRSA module, the FPRFN, and a lightweight grouped convolution module (GConv). The detailed experimental configurations are presented in Table 10, and the corresponding results are summarized in Table 11.

In the single-module experiments, the baseline model achieved an mAP@0.5 of 31.4% on the test set. The introduction of the DSAF module in Model1 improved the mAP@0.5 to 32.0%, demonstrating that this module effectively enhances the model’s feature representation capability by jointly extracting detailed features and semantic information. Model2, which solely incorporated the MSRSA module, attained an mAP@0.5 of 31.7%, indicating that the multi-scale spatial attention mechanism positively contributes to enhancing spatial perception and optimizing feature localization. By contrast, Model3 with only the FPRFN demonstrated more substantial improvement, elevating the mAP@0.5 to 34.8%. This significant enhancement further validates the critical role of shallow features in small object detection, where the fusion of multi-scale shallow information enables the model to better capture edge and texture characteristics of targets.

In the combined-module experiments, Model4, which incorporated both the DSAF and MSRSA modules, achieved an improved mAP@0.5 of 32.3%, demonstrating enhanced semantic modeling and spatial perception capabilities. Model5, combining DSAF with FPRFN showed further performance gains, reaching 35.1% in mAP@0.5. This was the best performance among all combinations and indicated significant complementary effects between semantic-detail fusion and shallow feature enhancement for small object detection. Both Model6 (MSRSA + FPRFN) and Model7 (DSAF + MSRSA + FPRFN) maintained stable performance improvements while keeping model complexity at a moderate level. The final Model7 attained an mAP@0.5 of 35.5%, representing a 4.1 percentage point improvement over the baseline model. These results conclusively demonstrate the effectiveness of multi-module collaborative optimization.

The final YOLO-DMF model was developed by integrating the lightweight GConv module into Model7. Although a slight decrease in accuracy was observed, this enhancement significantly reduced both the model parameters and computational complexity. The improved architecture exhibits superior lightweight characteristics while maintaining competitive detection performance, thereby substantially enhancing its potential for real-world deployment scenarios.

**Figure 7:**
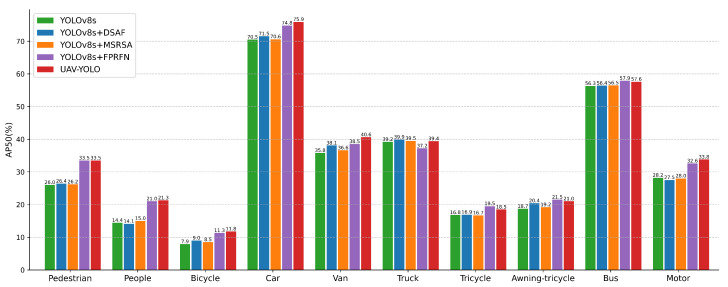
Comparison of detection accuracy for different improved modules across categories.

To provide a more detailed understanding of the individual contributions of each proposed module to model performance, we conducted an in-depth analysis of their detection capabilities across different object categories. The specific results are visualized in Figure 7. As clearly illustrated in Figure 7, each enhancement module demonstrates distinct performance improvements across different target categories. The detailed analysis reveals the following insights:

The DSAF module demonstrates particularly significant improvements in the detection of vehicle-related categories, including Car, Van, and Awning-tricycle, with performance gains of 1.0%, 2.3%, and 1.7%, respectively. These results suggest that the module excels in scenarios where both fine-grained feature extraction and high-level semantic understanding are required, highlighting its effectiveness for object classes that demand precise structural and contextual modeling.

The MSRSA module achieves the most notable performance improvements on the Van, People, and Bicycle categories with gains of 0.8%, 0.6%, and 0.6%, respectively. These results demonstrate its effectiveness in enhancing spatial perception and improving feature localization, thereby indicating its strong suitability for object detection tasks involving targets with distinct motion characteristics.

The FPRFN achieves the most significant improvements on small-scale objects such as Pedestrian and People with performance gains of 7.5% and 6.6%, respectively. This result underscores the critical role of integrating multi-scale shallow features in enhancing detection performance for small targets.

Building upon the integration of all improved modules, YOLO-DMF achieves a well-balanced performance improvement across all object categories. Notably, it demonstrates superior performance in handling challenging classes such as Bicycle and Tricycle, with mAP@0.5 improvements of 3.9% and 1.7%, respectively, on the test set. These results clearly highlight the benefits of multi-module collaborative optimization and underscore the effectiveness of the proposed approach in improving detection accuracy in complex scenarios.

### 4.5. Visual Analysis

To more comprehensively evaluate the detection performance of YOLOv8s and YOLO-DMF under varying small-object distributions, this study evaluates the models through comparative experiments across three representative complex scenarios: densely versus sparsely distributed targets, occluded versus non-occluded environments, and low-light versus high-light illumination conditions. These scenarios represent typical yet challenging situations commonly encountered in object detection tasks based on UAV platforms.

**Figure 8:**
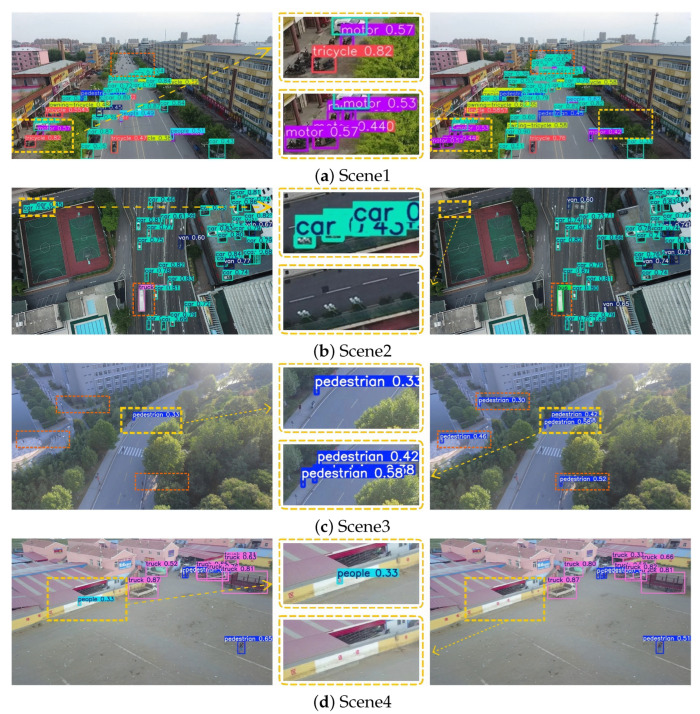
Comparison of detection results in dense and sparse target distribution scenarios.

**Figure 9:**
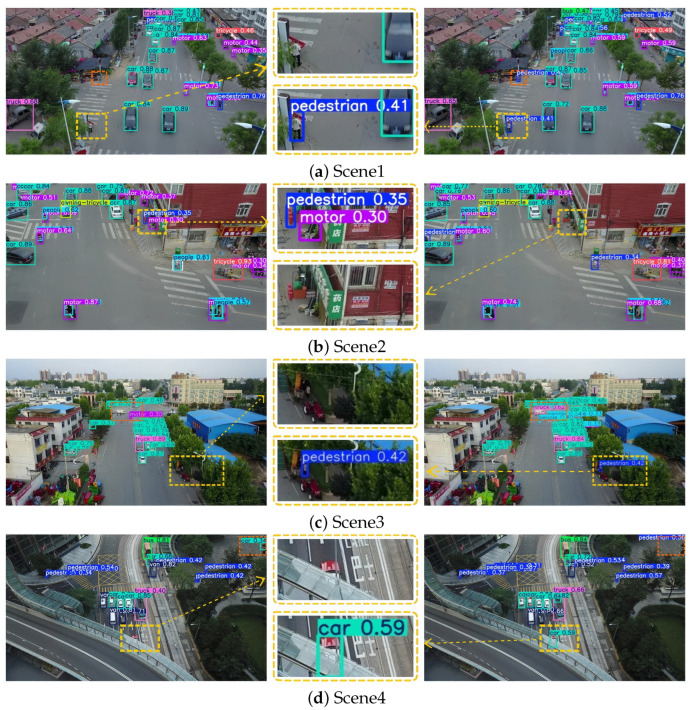
Comparison of detection results in occluded and non-occluded scenarios.

**Figure 10:**
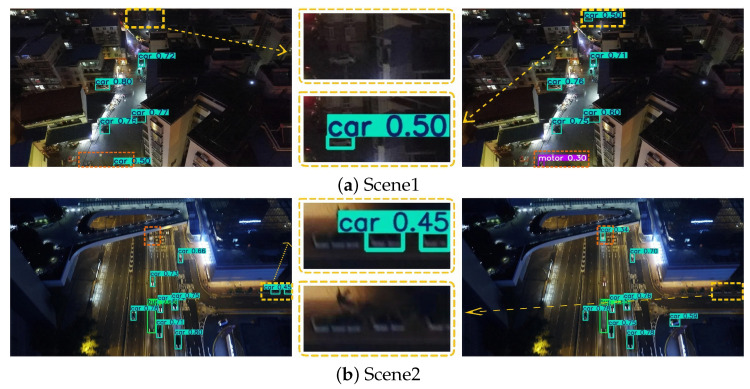

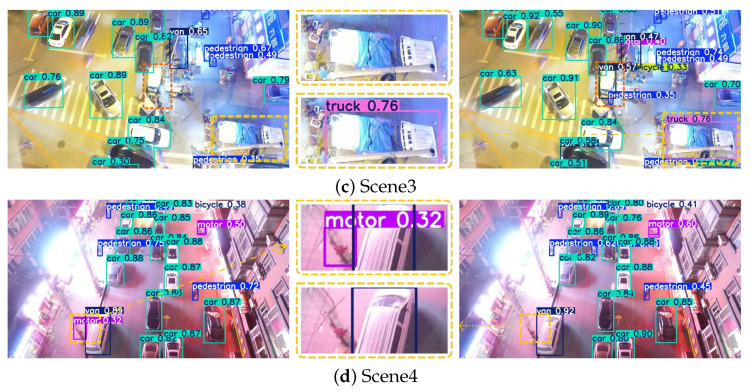
Visual comparison of detection results in low-light and high-light scenarios.

Specifically, Figure 8 illustrates the detection results under dense and sparse target distributions; Figure 9 presents the performance differences between the two models in occluded and non-occluded conditions; and Figure 10 displays their detection effectiveness under varying illumination environments. In each set of comparison images, the left side shows the detection visualization result of the YOLOv8s model, while the right side corresponds to that of YOLO-DMF. Regions with discrepancies in detection results, such as missed or incorrect detections, are marked with dashed bounding boxes. Additionally, a magnified view of a representative discrepancy region is provided in the central part of each figure, allowing for a more detailed visual comparison of the models’ performance in key regions.

As shown in Figure 8, in Scenario 1, YOLO-DMF achieves a more accurate detection of motorcycles parked near storefronts and clusters of distant cars. In Scenario 2, YOLOv8s erroneously identifies streetlights along the stadium perimeter as cars and misclassifies a moving bus as a truck, whereas YOLO-DMF achieves accurate detection. In Scenario 3, YOLOv8s suffers from a high rate of missed detections for pedestrians. In Scenario 4, markings on the outer wall of the parking area are falsely detected as groups of people by YOLOv8s, whereas YOLO-DMF effectively avoids such false positives. These results collectively demonstrate that YOLO-DMF consistently outperforms YOLOv8s in detecting small objects under both dense and sparse distributions.

As shown in Figure 9, in Scenario 1, two pedestrians at the end of a zebra crossing were not detected by YOLOv8s. In Scenario 2, objects placed in front of a store entrance were falsely identified as a pedestrian and a motorcycle by YOLOv8s. In contrast, in Scenario 3, despite partial occlusion caused by tree shade, YOLO-DMF successfully detected the pedestrian and accurately recognized the truck at the urban intersection. In Scenario 4, YOLO-DMF achieved a more precise localization of an occluded car beneath elevated structures while maintaining a low false positive rate in distant background regions such as flower beds. Overall, these experimental results indicate that YOLO-DMF exhibits significant advantages over YOLOv8s in detecting small objects under complex conditions involving occlusions.

As shown in Figure 10, YOLOv8s exhibits a considerable number of false positives and missed detections across various real-world scenarios. Specifically, in Scenario 1, the model fails to correctly identify a moving motorcycle and a distant car under low-light nighttime conditions. In Scenario 2, it erroneously detects two normally moving objects on the road as cars and misclassifies roadside flower beds as cars. In Scenario 3, under favorable lighting conditions, the model misses the detection of a truck parked at the roadside. In Scenario 4, parts of a utility pole near the road are falsely detected as a motorcycle. These results indicate that YOLOv8s still faces certain limitations in detecting small objects under varying illumination conditions and complex background interference, whereas YOLO-DMF demonstrates superior robustness and adaptability.

### 4.6. Comparative Study

To comprehensively evaluate the small object detection performance of the YOLO-DMF model in UAV applications, we conducted a comparative analysis against several mainstream lightweight models from the YOLO series, which were evaluated on three representative aerial datasets: VisDrone2019, WAID, and AI-TOD. Additionally, comparisons with state-of-the-art specialized small object detection algorithms were performed exclusively on the VisDrone2019 dataset to assess the most advanced performance in this domain. All experiments were implemented using the unified YOLO framework provided by Ultralytics. To ensure a fair comparison and reliable results, all models were trained under identical experimental conditions until full convergence.

To comprehensively evaluate the small object detection performance of the YOLO-DMF model in UAV-based applications, we conducted a comparative analysis against several mainstream lightweight models from the YOLO series as well as state-of-the-art small object detection algorithms. All experiments were implemented using the unified YOLO framework provided by Ultralytics and evaluated on three representative aerial datasets: VisDrone2019, WAID, and AI-TOD. To ensure a fair comparison and reliable results, all models were trained under identical experimental conditions until full convergence.

**Figure 11:**
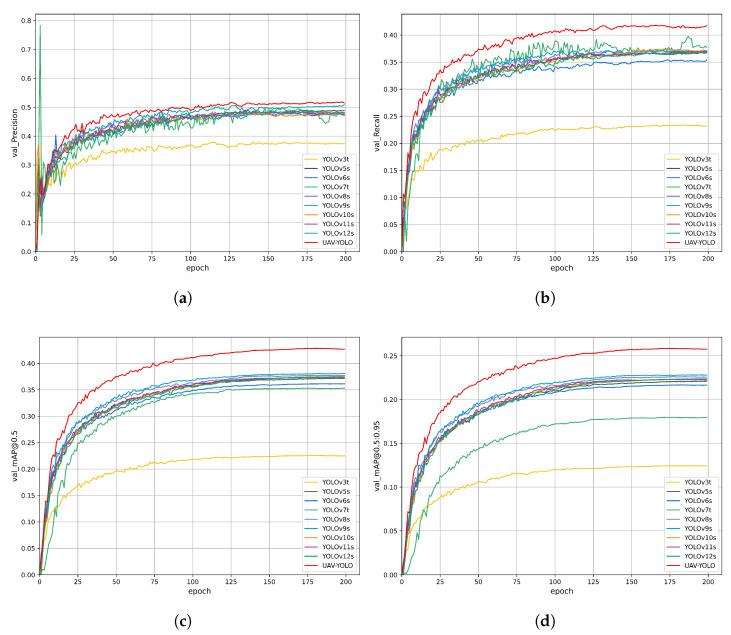
Training and validation metrics curves of the YOLO-DMF model. (**a**) Training loss curve. (**b**) Validation loss curve. (**c**) Validation set mAP@0.5 curve. (**d**) Validation set mAP@0.5:0.95 curve.

To verify the fast convergence and high convergence accuracy of the YOLO-DMF model, we conducted a comprehensive comparative analysis between YOLO-DMF and several lightweight object detection models on the VisDrone2019 dataset. The complete comparison results are presented in Figure 11. As shown in Figure 11, YOLO-DMF demonstrates significant advantages in network convergence speed, achieving higher accuracy in fewer training epochs compared to other models. Furthermore, YOLO-DMF shows notable performance improvements across all evaluation metrics. Detailed experimental results are presented in Table 12.

#### 4.6.1. Performance Comparison on the VisDrone2019 Dataset

As shown in Table 12, YOLO-DMF achieves state-of-the-art detection accuracy among mainstream lightweight models while maintaining an efficient architecture with only 3.7M parameters. Although the computational cost marginally increases to 29.3 GFLOPs, the model delivers substantial performance improvements, achieving an mAP@0.5 of 35.3% and an mAP@0.5:0.95 of 20.6%, respectively—representing increases of 3.9 and 2.5 percentage points over the baseline model. These results demonstrate YOLO-DMF’s superior suitability for precision-sensitive edge computing applications, where it effectively balances real-time processing requirements with enhanced detection performance. Moreover, the model achieves a core inference speed of 34.1 FPS on the RK3588 NPU, demonstrating sufficient computational efficiency to support high-frame-rate real-time detection systems.

**Figure 12:**
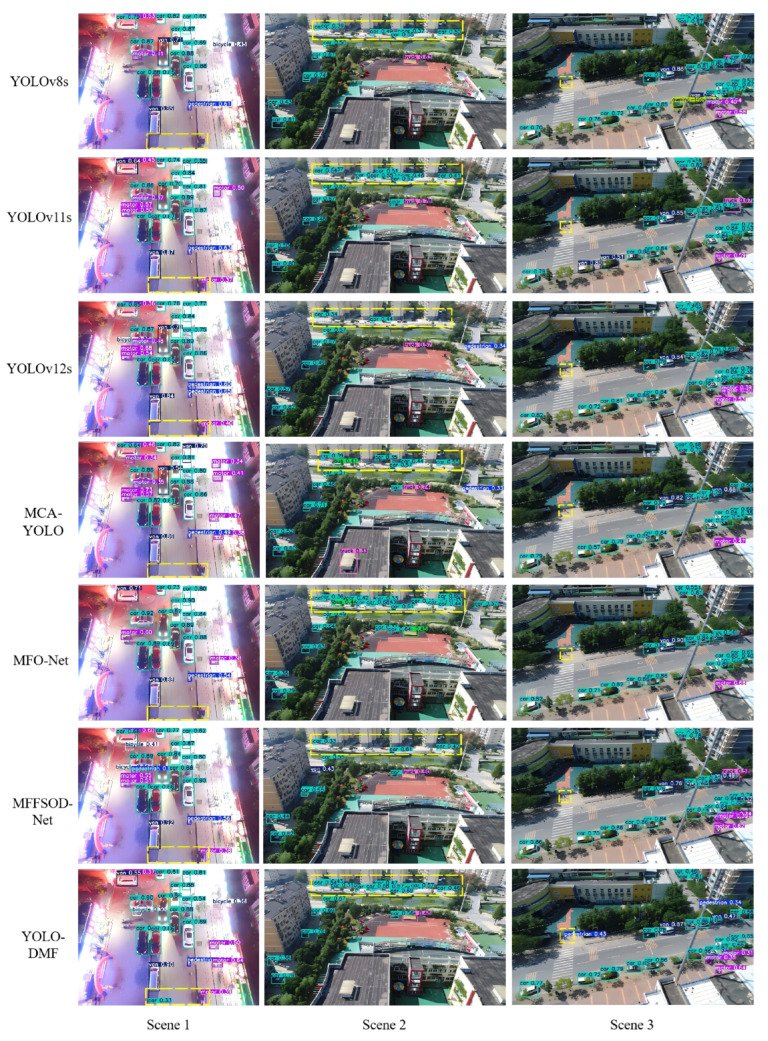
Visualization comparison of YOLO-DMF with various state-of-the-art algorithms across multiple scenarios.

**Figure 13:**
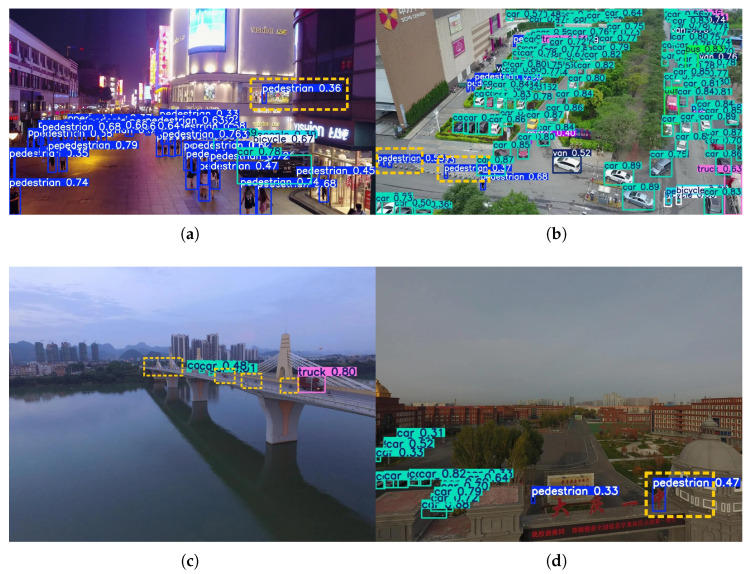
Visualization of representative false positives and missed detections by YOLO-DMF. (**a**) Scene 1. (**b**) Scene 2. (**c**) Scene 3. (**d**) Scene 4.

To further validate the model performance, we compared the visual detection results of YOLO-DMF with those of several state-of-the-art algorithms under challenging scenarios, including dense scenes in both daytime and nighttime conditions, as well as scenes containing tiny objects, as illustrated in Figure 12. Although YOLO-DMF achieved significantly improved detection accuracy on the VisDrone2019 dataset and effectively addressed most existing detection challenges, it still faces certain limitations in practical applications due to the inherent difficulty of detecting small objects in drone-captured imagery. As shown in Figure 13, YOLO-DMF still suffers from varying degrees of false positives and missed detections in complex scenarios.

As shown in Figure 12, YOLO-DMF demonstrates superior robustness, achieving higher recall across all three evaluated scenarios and exhibiting particularly strong performance in detecting small objects. Nevertheless, as illustrated in Figure 13, the model still suffers from false positives and missed detections in certain complex scenes. Specifically, in Scenario 1, a mannequin inside a shopping mall is erroneously detected as a pedestrian; in Scenario 2, cone-shaped traffic barriers along the roadside are misclassified as pedestrians; in Scenario 3, numerous distant vehicles on a bridge are missed due to their extremely small scale; and in Scenario 4, a school nameplate is mistakenly identified as a pedestrian. These cases highlight the ongoing challenges in distinguishing small or atypical objects from true foreground targets under visually cluttered conditions.

A comprehensive analysis indicates that the primary cause of these false positives lies in the high visual similarity between the detected objects and the target category, reflecting the need for further improvement in the model’s semantic understanding capability. To mitigate such false positives caused by visual ambiguity, future work will explore the integration of fine-grained semantic alignment mechanisms or class-discriminative feature enhancement modules to strengthen the model’s ability to distinguish between visually similar categories. Moreover, small targets often appear blurred and with low resolution in drone-captured images, which further highlights the importance of large receptive field design in small-object detection. A larger receptive field enables the model to capture more contextual information, thereby enhancing its overall discriminative capability.

#### 4.6.2. Performance Comparison on the WAID Dataset

The comparative results of the previously mentioned state-of-the-art algorithms are summarized in Table 13. These results were obtained through experiments conducted on the WAID dataset. As shown in Table 13, with model parameters and computational cost comparable to those of other mainstream lightweight detectors, YOLO-DMF achieves significantly better performance on the WAID dataset than the baseline YOLOv8s model. Specifically, YOLO-DMF obtains mAP@0.5 and mAP@0.5:0.95 scores of 96.4% and 62.8%, representing improvements of 1.1 and 1.7 percentage points, respectively. These results confirm the effectiveness of YOLO-DMF on the WAID dataset and highlight its potential for practical deployment in real-world wildlife monitoring applications.

#### 4.6.3. Performance Comparison on the AI-TOD Dataset

Similarly, Table 14 presents the comparative results of different detection algorithms on the AI-TOD dataset. The experimental results in Table 14 demonstrate YOLO-DMF’s strong generalization capability on the AI-TOD dataset, achieving a state-of-the-art mAP@0.5 of 43.6% among all compared lightweight detectors. This corresponds to a substantial improvement of 4.0 percentage points over the baseline YOLOv8s model, which achieves 39.6% mAP@0.5. These findings provide robust evidence for YOLO-DMF’s superior detection performance in challenging aerial scenarios characterized by dense small object distributions and significant scale variations.

## 5. Conclusions

Small object detection in UAV imagery is hindered by limited feature representation, complex background clutter, and extremely small target scales, which degrade the accuracy and efficiency of conventional detectors—particularly in real-world applications. To address these limitations, we propose YOLO-DMF, which is a purpose-built framework incorporating three targeted innovations. First, the DSAF module establishes a multi-branch architecture to harmonize shallow detail features with deep semantic cues, thereby enriching feature expressiveness and mitigating the semantic-detail imbalance inherent in deep networks. Second, the MSRSA mechanism employs scale-aware spatial attention to dynamically enhance responses in target regions while suppressing activations from noisy backgrounds, improving model robustness against clutter. Third, the FPRFN reconstructs the feature hierarchy through cross-level integration, reusing high-resolution shallow features and hierarchically fusing them with deep semantics, further enhanced by a dedicated 160 × 160 detection head for the precise localization of tiny objects. Together, these components form a cohesive and efficient architecture specifically tailored to the unique challenges of aerial small object detection. Experimental results on benchmark datasets—including VisDrone2019, WAID, and AI-TOD—demonstrate the effectiveness and strong generalization capability of the proposed method.

YOLO-DMF rethinks the architecture of aerial small object detection by prioritizing contextual continuity and hierarchical feature equity—a design philosophy that challenges the conventional marginalization of shallow features. Instead of discarding high-resolution details in pursuit of semantic abstraction, our framework establishes a bidirectional synergy between deep semantics and early spatial cues, ensuring that fine-grained information is preserved, refined, and strategically reintegrated into the detection pipeline.

Despite its strong performance on benchmark datasets, several limitations remain. The model’s accuracy may degrade under extreme weather conditions (e.g., heavy fog, rain), motion blur, or severe occlusion—scenarios common in real-world UAV deployments but underrepresented in current training data. This highlights the need for improved robustness through advanced data augmentation, domain adaptation, or uncertainty-aware modeling. These issues point to promising directions for future work, including robust perception under adverse conditions and energy-efficient inference for edge deployment.

Future work will focus on model compression techniques—such as structured pruning and knowledge distillation—to improve deployment efficiency on resource-constrained platforms, and we plan to extend YOLO-DMF to video-based detection and tracking frameworks to enable continuous monitoring in time-critical UAV missions. Recognizing the current limitations in handling extreme environmental conditions—such as heavy fog, intense rain, or severe occlusion—we will further investigate robust perception strategies tailored for such adverse scenarios. Specifically, we aim to integrate multi-spectral sensing cues (e.g., thermal or near-infrared data) and incorporate uncertainty-aware feature refinement mechanisms to enhance model reliability under low-visibility or partially observable conditions. Ultimately, this work contributes not only a high-performance detector but also a principled deep network design paradigm for aerial vision, emphasizing contextual continuity and hierarchical feature equity.

## Figures and Tables

**Table 1:** Details of the proposed YOLO-DMF network. Layers denotes the module index, From indicates the input source index, and N specifies the number of times the module is stacked.

Components	Layers	From	N	Params	Module	Arguments
Backbone	0	−1	1	12,496	DSAF	[3, 32]
	1	−1	1	704	GCBS	[32, 64, 3, 2]
	2	−1	1	29,056	C2F	[64, 64, 1]
	3	−1	1	1408	GCBS	[64, 128, 3, 2]
	4	−1	1	115,456	C2F	[128, 128, 1]
	5	−1	1	2816	GCBS	[128, 256, 3, 2]
	6	−1	1	460,288	C2F	[256, 256, 1]
	7	−1	1	2816	GCBS	[256, 256, 3, 2]
	8	−1	1	460,288	C2F	[256, 256, 1]
	9	−1	1	164,608	SPPF	[256, 256, 5]
	10	−1	1	143,032	MSRSA	[256]
Neck	11	−1	1	0	Upsample	[None, 2, ‘nearest’]
	12	[−1, 6]	1	0	Concat	[1]
	13	−1	1	525,824	C2F	[512, 256, 1]
	14	−1	1	0	Upsample	[None, 2, ‘nearest’]
	15	[−1, 4]	1	0	Concat	[1]
	16	−1	1	148,224	C2F	[384, 128, 1]
	17	−1	1	0	Upsample	[None, 2, ‘nearest’]
	18	[−1, 2]	1	0	Concat	[1]
	19	−1	1	37,248	C2F	[192, 64, 1]
	20	−1	1	36,992	CBS	[64, 64, 3, 2]
	21	[−1, 4, 16]	1	0	Concat	[1]
	22	−1	1	140,032	C2F	[320, 128, 1]
	23	−1	1	147,712	CBS	[128, 128, 3, 2]
	24	[−1, 6, 13]	1	0	Concat	[1]
	25	−1	1	558,592	C2F	[640, 256, 1]
Head	26	−1	1	753,262	Detect	[10, [64, 128, 256]]
summary: 156 layers, 3,740,854 parameters, 3,740,838 gradients, 29.6 GFLOPs						

**Table 2:** Statistics of the VisDrone2019 dataset.

Types	Label	Number			
		Training Set	Validation Set	Testing Set	Total
Pedestrian	0	79,336	8843	21,005	109,184
People	1	27,058	5124	6375	38,557
Bicycle	2	10,479	1286	1301	13,066
Car	3	144,866	14,063	28,073	187,002
Van	4	24,955	1974	5770	32,699
Truck	5	12,874	749	2658	16,281
Tricycle	6	4811	1044	529	6384
Awning-tricycle	7	3245	531	598	4374
Bus	8	5952	250	2939	9114
Motor	9	29,646	4885	5844	40,375

**Table 3:** Statistics of the WAID dataset.

Types	Label	Number			
		Training Set	Validation Set	Testing Set	Total
Sheep	0	91,495	26,062	13,322	130,879
Cattle	1	44,244	12,604	6239	63,087
Seal	2	15,761	4925	2688	23,374
Camelus	3	4675	1270	675	6620
Kiang	4	3311	836	459	4606
Zebra	5	3791	1000	431	5222

**Table 4:** Statistics of the AI-TOD dataset.

Types	Label	Number			
		Training Set	Validation Set	Testing Set	Total
Airplane	0	622	169	744	1535
Bridge	1	511	139	688	1338
Storage-tank	2	5268	2476	5859	13,603
Ship	3	13,538	3790	17,632	34,960
Swimming-pool	4	292	33	291	616
Vehicle	5	248,041	59,903	306,664	614,608
Person	6	14,125	3840	15,442	33,407
Windmill	7	175	66	289	530

**Table 5:** Environment configuration.

Environment Item	Configuration Parameters
Operating System	CentOS 8.5.2
GPU NVIDIA GeForce RTX 5090	32 G
CUDA	12.0
CPU	Intel(R) Xeon(R) Platinum 8336C
Memory	314G
Deep Learning Framework	PyTorch
Programming Language	Python3.9

**Table 6:** Training parameters.

Hyperparameter	Value
Image size	640 × 640
Batch size	64
Epoch	200
Learning rate	0.01
Optimizer	SGD
Momentum	0.937
Weight decay	0.0005

**Table 7:** Performance and complexity of different attention modules.

Model	P (%)	R (%)	mAP@0.5 (%)	mAP@0.5:0.95 (%)	Params ($10^{6}$)	GFLOPs
YOLOv8s	43.3	33.2	31.4	18.1	11.1	28.5
+SEv1 [31]	45.3	32.7	31.3	18.1	11.2	28.5
+SEv2 [32]	42.6	33.0	31.2	18.0	11.2	28.5
+ECA [25]	44.0	32.5	31.0	17.9	11.1	28.5
+SimAM [33]	43.3	32.7	31.1	18.0	11.1	28.5
+CBAM [26]	44.4	32.7	31.5	18.1	11.4	28.7
+CA [34]	44.9	32.9	31.5	18.2	11.2	28.5
+GAM [16]	42.7	33.1	31.3	18.1	17.7	33.7
+EMA [35]	43.1	33.0	31.0	17.9	11.1	28.5
+SKA [36]	44.3	32.6	31.2	18.1	33.2	46.1
+MSRSA (ours)	44.1	33.5	31.7	18.3	11.7	28.9

**Table 8:** Comparison experiment of shallow feature layer fusion methods.

Model	P (%)	R (%)	mAP@0.5 (%)	mAP@0.5:0.95 (%)	Params ($10^{6}$)	GFLOPs
PAN- P3+P4+P5	43.3	33.2	31.4	18.1	11.1	28.5
PAN- P2+P3+P4	45.4	36.2	34.5	19.9	7.4	34.1
PAN- P2+P3+P4+P5	45.6	35.8	34.2	19.8	10.6	36.7
FPRFN- P2+P3+P4 (ours)	46.7	35.8	34.8	20.0	7.5	34.5

**Table 9:** Comparison of shallow feature layer fusion methods on small, medium, and large targets.

Model	${\mathrm{AP}}_{S}$ (%)	${\mathrm{AP}}_{M}$ (%)	${\mathrm{AP}}_{L}$ (%)
PAN-P3+P4+P5	7.3	26.0	37.5
PAN-P2+P3+P4	9.2 (+1.9)	27.4 (+1.4)	36.9 (−0.6)
PAN-P2+P3+P4+P5	9.2 (+1.9)	27.4 (+1.4)	38.3 (+0.8)
FPRFN-P2+P3+P4 (ours)	9.4 (+2.1)	27.4 (+1.4)	38.2 (+0.7)

**Table 10:** Experimental configuration for ablation study.

Model	DSAF	MSRSA	FPRFN	GConv
Baseline	×	×	×	×
Model1	√	×	×	×
Model2	×	√	×	×
Model3	×	×	√	×
Model4	√	√	×	×
Model5	√	×	√	×
Model6	×	√	√	×
Model7	√	√	√	×
YOLO-DMF	√	√	√	√

**Table 11:** Experimental results of ablation study.

Model	P (%)	R (%)	mAP@0.5 (%)	mAP@0.5:0.95 (%)	Params ($10^{6}$)	GFLOPs
Baseline	43.3	33.2	31.4	18.1	11.1	28.5
Model1	44.8	34.1	32.0	18.6	11.1	30.6
Model2	44.3	33.4	31.7	18.3	11.7	28.9
Model3	46.7	35.8	34.8	20.0	7.5	34.5
Model4	43.4	34.0	32.3	18.7	11.7	31.0
Model5	46.7	35.9	35.1	20.4	7.5	36.7
Model6	46.5	35.5	34.9	20.2	8.0	35.0
Model7	47.6	36.5	35.5	20.4	8.0	37.1
YOLO-DMF	47.3	36.0	35.3	20.6	3.7	29.3

**Table 12:** Comparison results of different algorithms on the Visdrone2019 dataset.

Model	P (%)	R (%)	mAP@0.5 (%)	mAP@0.5:0.95 (%)	Params ($10^{6}$)	GFLOPs	FPS
YOLOv3t [37]	34.3	20.6	18.8	10.2	12.1	18.9	57.2
YOLOv5s [38]	42.2	32.6	30.7	17.7	9.1	23.8	48.4
YOLOv6s [39]	42.4	31.3	30.0	17.5	16.3	44.0	36.4
YOLOv7t [40]	41.7	33.8	29.4	14.5	6.0	13.1	72.7
YOLOv8s [41]	43.3	33.2	31.4	18.1	11.1	28.5	38.0
YOLOv9s [42]	45.8	33.1	31.8	18.4	7.2	26.7	29.4
YOLOv10s [43]	43.9	32.9	31.4	18.0	8.0	24.5	33.8
YOLOv11s [44]	42.8	33.2	31.0	17.9	9.4	21.3	33.0
YOLOv12s [45]	43.5	33.0	31.4	18.1	9.2	21.2	31.3
MCA-YOLO [46]	41.1	34.4	30.8	16.3	6.3	12.4	73.6
MFO-Net [47]	43.2	34.5	31.4	17.8	6.4	12.5	72.4
MFFSODNet [48]	39.6	33.2	30.1	16.0	1.6	21.5	50.2
YOLO-DMF	47.3	36.0	35.3	20.6	3.7	29.3	34.1

**Table 13:** Comparison results of different algorithms on the WAID dataset.

Model	P (%)	R (%)	mAP@0.5 (%)	mAP@0.5:0.95 (%)	Params ($10^{6}$)	GFLOPs	FPS
YOLOv3t [37]	90.3	77.9	85.4	49.2	12.1	18.9	57.2
YOLOv5s [38]	92.8	92.0	95.1	60.3	9.1	23.8	48.4
YOLOv6s [39]	93.1	90.3	94.8	59.8	16.3	44.0	36.4
YOLOv7t [40]	94.4	90.7	95.3	56.1	6.0	13.1	72.7
YOLOv8s [41]	94.0	92.2	95.3	61.0	11.1	28.4	38.0
YOLOv9s [42]	94.3	92.8	96.0	61.8	7.2	26.7	29.4
YOLOv10s [43]	93.9	92.0	95.9	61.4	8.0	24.5	33.8
YOLOv11s [44]	94.2	91.5	95.3	61.2	9.4	21.3	33.0
YOLOv12s [45]	93.6	92.0	95.5	61.1	9.2	21.2	31.3
YOLO-DMF	94.3	93.3	96.4	62.8	3.7	29.3	34.1

**Table 14:** Comparison results of different algorithms on the AI-TOD dataset.

Model	P (%)	R (%)	mAP@0.5 (%)	mAP@0.5:0.95 (%)	Params ($10^{6}$)	GFLOPs	FPS
YOLOv3t [37]	37.9	20.9	20.9	8.6	12.1	18.9	57.2
YOLOv5s [38]	52.5	38.1	38.7	17.1	9.1	23.8	48.4
YOLOv6s [39]	70.3	32.7	34.0	15.2	16.3	44.0	36.4
YOLOv7t [40]	73.0	28.1	27.1	10.6	6.0	13.2	72.7
YOLOv8s [41]	53.0	38.7	39.6	17.5	11.1	28.5	38.0
YOLOv9s [42]	58.4	38.4	39.1	17.5	7.2	26.7	29.4
YOLOv10s [43]	65.3	36.6	38.8	17.4	8.0	24.5	33.8
YOLOv11s [44]	52.4	37.0	38.7	17.2	9.4	21.3	33.0
YOLOv12s [45]	48.3	27.0	28.4	12.4	9.2	21.2	31.3
YOLO-DMF	63.0	41.5	43.6	19.5	3.7	29.2	34.1

## Data Availability

The original contributions presented in this study are included in the article. Further inquiries can be directed to the corresponding author.
